# Commentary: Are groups more or less than the sum of their members? The moderating role of individual identification

**DOI:** 10.3389/fpsyg.2018.00999

**Published:** 2018-06-15

**Authors:** Zhonglu Zhang, Christopher M. Warren, Yi Lei, Qiang Xing, Hong Li

**Affiliations:** ^1^Department of Psychology, Guangzhou University, Guangzhou, China; ^2^Research Centre for Brain Function and Psychological Science, Shenzhen University, Shenzhen, China; ^3^Department of Psychology, Utah State University, Logan, UT, United States; ^4^Shenzhen Institute of Neuroscience, Shenzhen, China; ^5^Institute of Affective and Social Neuroscience, Shenzhen University, Shenzhen, China

**Keywords:** individuation, shared identity, culture, task characteristics, group success

Baumeister et al. (2016) proposed that people perform better in groups only “when members of the group are individually identified and responsible” (p. 2), and conversely, that people perform worse in groups when they “are not publicly identified or rewarded” (p. 2). In other words, they emphasized how individual responsibility contributes to group success. However, we argue that shared identity, whereby group members share a common responsibility, can also facilitate group success in many circumstances, and thus should not be discounted. Several authors have shared the same view in the open peer commentary published in *Behavioral and Brain Sciences* about the ideas of Baumeister et al. (e.g., Budescu and Maciejovsky, 2016; Haslam and Ellemers, 2016; Nijstad and De Dreu, 2016). These authors had noted the special role of shared identity or having a common goal in facilitating bonds between members. Consistent with this, Ein-Dor and Hirschberger (2016) show how forming a cohesive group is a prerequisite for whether differentiation can have its maximal effect on group success. We argue two additional factors ignored by Baumeister et al. influence the effect of individual identity on group success: cultural differences and task characteristics.

We hold that the ideas of Baumeister et al. do not generalize beyond individualistic cultures, and do not generalize to all kinds of tasks. We begin by describing important differences between individualistic and collectivist cultures. Later, we distinguish between survival and development tasks. Ultimately, we argue that the benefit of individuation in groups may only apply to a small portion of the space defined by individualistic vs. collectivist cultures, and survival vs. development tasks, as schematically depicted in Figure 1. By contrast, the benefit of shared identity applies to any survival task, and to any task undertaken within a collectivist culture.

**Figure 1:**
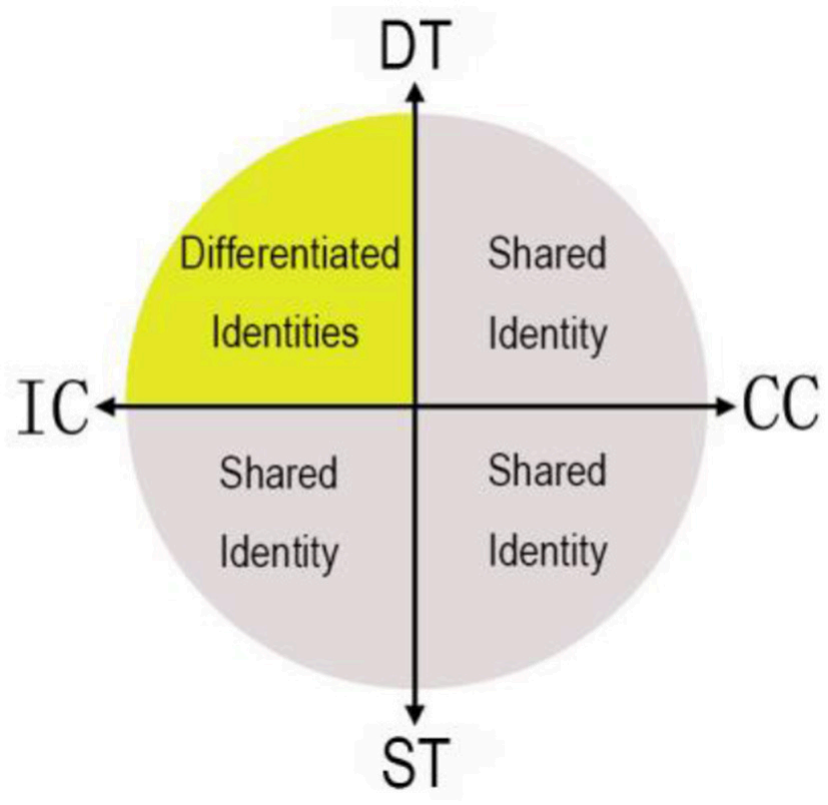
The benefit of differentiated identity over shared identity to group success is limited to specific tasks and cultures. ST denotes survival tasks; DT denotes developmental tasks; IC denotes individualistic culture; CC denotes collectivistic culture. The benefit of differentiated identities (vs. shared identity) is confined to developmental tasks in individualistic cultures (yellow area).

Baumeister et al. (2016) ignore culture as a factor that influences how people perform in groups. Previous work has highlighted the prominent differences between individualistic and collectivistic cultures where self-identity is concerned: whereas individual identity is emphasized in individualistic cultures, shared identity is emphasized in collectivistic cultures. According to the self-construal theory of individualism and collectivism (Triandis et al., 1988; Markus and Kitayama, 1991; Chiao et al., 2009; Saad et al., 2015), there are basic differences in how people construe themselves and their relationships between individualistic and collectivistic cultures. For example, in a task where subjects are supposed to draw themselves within a group, members of individualistic cultures draw themselves bigger than others in the group, whereas members of collectivist cultures draw themselves equal or even smaller than others in the group (Kitayama et al., 2009; Talhelm et al., 2014). Generally, people in individualistic cultures have independent self-construal such as autonomy and distinctiveness, and competitiveness/conflict is encouraged. In contrast, people in collectivistic cultures have interdependent self-construal that is largely defined by their surrounding context and relationships: sharing, cooperation, and group harmony are most important. These differences are especially relevant to group dynamics and relationships within groups. For example, reaction to honesty or deception is tempered by the intimacy of the relationship more so in collectivist cultures than in individualistic cultures (Wang et al., 2011). Neural data also supports differences in self-construal between collectivist and individualistic cultures. Collectivists have a shared neural representation of self and close family members (e.g., mother) whereas individualists do not (Zhu et al., 2007), In addition, Chiao et al. (2009) showed that medial prefrontal cortex activation is greater for collectivists in response to contextual self-description (shared identity), but is greater for individualists in response to general self-description (individual identity).

We agree with the view that for many types of tasks, the promotion of individual identity can facilitate group success, whereas promoting shared identity can reduce success. However, we argue that this result applies most strongly to individualistic cultures, and may not be observed at all when testing collectivist cultures (also pointed out by Brown, 2016). More crucially, we argue that the role of perceived identity in group success is modulated by culture. Specifically, the promotion of individual identity can facilitate group success for individualists but not collectivists. In contrast, the promotion of shared identity can bolster group work for collectivists but not for individualist. There is ample evidence for this position. First, individualists and collectivists show the opposite pattern of performance when working in groups vs. when working alone. For example, individualists are more likely to exhibit social loafing (exerting less effort when in a group) whereas collectivists are more likely to exert more effort in a group (Earley, 1989, 1993; Karau and Williams, 1993; Wagner, 1995). Second, but more important, the effects of individualism on group performance is typically driven by criticism, competitiveness, and the desire to be unique, whereas the effect of collectivism on group performance is driven by cooperation (Tjosvold et al., 2003; Goncalo and Staw, 2006). As such, recent evidence shows that priming collectivist values facilitates team ideation by promoting cooperation (Ye and Robert, 2017). Furthermore, criticism improves performance on creative brainstorming tasks for members of individualistic cultures (Nemeth et al., 2004; Saad et al., 2015), but criticism does not benefit members of collectivist cultures (Saad et al., 2015).

Baumeister et al. (2016) also ignore how task characteristics can modulate performance in groups. We note a critical distinction between survival tasks and development tasks. Survival tasks refer to the struggle to remain living by securing sufficient food and shelter, and avoiding predators etc. (Buss, 2008). In terms of group goals, survival tasks are tasks directed at preventing the group from shrinking. In contrast, development tasks are directed at making the group thrive and grow. By our definition, the sorts of creativity tasks reviewed by Baumeister et al. constitute development tasks. However, Baumeister et al. implicitly generalize their ideas to any type of task directed toward group goals. They do allude to Tuckman's (1965) “group forming” stage, in which the benefits of shared group identity are highlighted, but Baumeister et al. (2016) diminish the importance of these findings as they relate to group success, relegating them to the “aegis of social identity theory” (p. 3). They go on to espouse the benefit of group differentiation, implicitly linking it to all other circumstances, ignoring that survival tasks are a central feature in the evolutionary history of humankind (Buss, 2008).

The probability of survival is increased by shared identity because shared identity promotes helping (Penner et al., 2005), loyalty (Van Vugt and Hart, 2004), contribution (De Cremer et al., 2008), and resource sharing (Tyler and Degoey, 1995), all of which facilitate group success for survival tasks. In contrast, development is essential for thriving beyond mere survival. Development requires diversity and differentiation. Most of the tasks addressed by Baumeister et al. (2016) do not involve survival tasks and thus can be labeled as development tasks. We agree that individuation largely facilitates group success for development tasks such as group performance on creativity tasks. In this vein, we argue that differentiated identities can contribute to group success for developmental tasks whereas shared identity has a larger influence on group success for survival tasks.

In short, Baumeister et al. (2016) have provided a valuable framework for understanding the way individuals contribute to group performance. We argue that the perceived benefit of individual vs. shared identity to group success is dependent on culture and task characteristics. We hope our argument contributes to a broader perspective on this issue.

## Author contributions

ZZ drafted the manuscript. CW provided revisions and language editing. YL, QX, and HL provided revisions.

### Conflict of interest statement

The authors declare that the research was conducted in the absence of any commercial or financial relationships that could be construed as a potential conflict of interest.
